# Putting the C Back into the ABCs: A Multi-Year, Multi-Region Investigation of Condom Use by Ugandan Youths 2003–2010

**DOI:** 10.1371/journal.pone.0093083

**Published:** 2014-04-04

**Authors:** Joseph J. Valadez, Caroline Jeffery, Rosemary Davis, Joseph Ouma, Stephen K. Lwanga, Sarah Moxon

**Affiliations:** 1 Liverpool School of Tropical Medicine, Department of International Public Health, Monitoring and Evaluation Technical assistance and Research Group, Liverpool, United Kingdom; 2 Management Sciences for Health, Kampala, Uganda; 3 Strengthening TB and AIDS Response in the Eastern Region - Lot Quality Assurance Sampling (STAR E-LQAS) Project, Kampala, Uganda; Columbia University, United States of America

## Abstract

A major strategy for preventing transmission of HIV and other STIs is the consistent use of condoms during sexual intercourse. Condom use among youths is particularly important to reduce the number of new cases and the national prevalence. Condom use has been often promoted by the Uganda National AIDS Commission. Although a number of studies have established an association between condom use at one’s sexual debut and future condom use, few studies have explored this association over time, and whether the results are generalizable across multiple locations. This multi time point, multi district study assesses the relationship between sexual debut and condom use and consistent use of condoms thereafter. Uganda has used Lot Quality Assurance Sampling surveys since 2003 to monitor district level HIV programs and improve access to HIV health services. This study includes 4518 sexually active youths interviewed at five time points (2003–2010) in up to 23 districts located across Uganda. Using logistic regression, we measured the association of condom use at first sexual intercourse on recent condom usage, controlling for several factors including: age, sex, education, marital status, age at first intercourse, geographical location, and survey year. The odds of condom use at last intercourse, using a condom at last intercourse with a non-regular partner, and consistently using a condom are, respectively, 9.63 (95%WaldCI = 8.03–11.56), 3.48 (95%WaldCI = 2.27–5.33), and 11.12 (95%WaldCI = 8.95–13.81) times more likely for those individuals using condoms during their sexual debut. These values did not decrease by more than 20% when controlling for potential confounders. The results suggest that HIV prevention programs should encourage condom use among youth during sexual debut. Success with this outcome may have a lasting influence on preventing HIV and other STIs later in life.

## Introduction

In the past decade, the international public health response to the human immunodeficiency virus (HIV) epidemic has been strengthened. This is partly due to biomedical advances, such as the introduction of male circumcision as a viable prevention measure [1] and the expansion of antiretroviral (ARV) treatment options [2]. Despite the potential of these interventions to reduce rates of HIV transmission, they offer only partial protection from the sexual transmission of HIV, the main mode of transmission in Sub-Saharan Africa [3], where HIV persists as a major public health and development challenge. If used correctly and consistently, the male latex condom remains an important method available to individuals that can offer up to 95% protection from the sexual transmission of HIV [4]. Consistent use of condoms can also protect against most other sexually transmitted infections (STIs) [5] and offer protection from unwanted pregnancies. However, UNAIDS raised concerns about the low rates of condom use in African countries, particularly by youths, a population group especially vulnerable to HIV [3], [6].

Uganda was the first country in sub-Saharan Africa to reverse the adult prevalence of HIV in the 1990s [7]. However, the impressive initial reversal in the trend of HIV prevalence plateaued in Uganda. In 2009, HIV prevalence in Uganda was 7.3% [8] marking a slight increase from previous estimates. Many HIV experts are reluctant to credit the early reduction in HIV prevalence to one key message or intervention. Nevertheless, condom distribution and promotion of their use is critical component of Uganda’s HIV prevention strategy.

Recent investigations of the predictors of condom use have produced diverse findings [9]–[14]. Education level, economic status, and age were all positively correlated with condom use [11], [15]–[17]. Exposure to prevention messages, while positively associated with condom use, is substantially influenced by sex, economic status, age difference between partners, and the length of the relationship [6], [12], [18]–[20]. Knowledge that you yourself may be at risk of HIV, does not necessarily lead to using a condom [19].

Given that contextual and demographic factors may not easily be targeted through shorter-term public health interventions, researchers have analysed the experience of condom use in early sexual encounters. A recent study reports that people who use condoms in early sexual encounters are more likely to use condoms in later sexual encounters, and that a positive experience of condom use is a strong predictor of subsequent use [21]. This finding is pertinent to Uganda, as in many other countries [22], a person’s sexual debut frequently occurs before the age of 18 [23], [24]. If condom use is established at the earliest sexual encounter, individuals may be more likely to use a condom at their most recent sexual encounter and more likely to use condoms consistently with both regular and non-regular partners [10], [25]–[28]. Despite concerns that promoting condom use in young adults encourages sexual promiscuity, more recent evidence indicates the contrary. A multi-country study suggests there is no increase in the number of sexual partners between those who were exposed to condom promotion programs at a younger or an older age [29].

Our current study contributes to this literature through investigation of a unique database. We consider the effects of condom use by youths at their sexual debut over time using retrospective cross-sectional data. The data were collected over multiple time points, using multiple independent surveys carried out each year in a large number of different districts at each time point. This study analysed factors associated with several condom use measures in Ugandan youth aged 15–24 years old (n = 4493) over a 7-year period (2003–2010). We assess whether condom use at sexual debut was associated with recent condom use, adjusting for time, age, sex, education and Ugandan region. We cannot include economic status in this assessment, because household assets questions were not included in the questionnaires. The questions on condom use and sexual behaviour remained unchanged across the study time period. We carried out this epidemiological study with the aim of understanding determinants of condom use in Ugandan youth, over time, in order to inform future prevention programs. We hypothesised that those youth using a condom at sexual debut would be more likely to use a condom consistently and at future sexual encounters, adjusting for a number of demographic factors, such as age and educational level, in addition to time and space. We also expected that this relationship would exist regardless of the type of partner (regular or non-regular) and perceived risk of HIV. If messages and experiences learned in early sexual encounters play a consistently strong role in shaping future behaviours related to safer sex, our results could offer important evidence to guide social policy towards more focused and targeted behavioural interventions to successfully promote condom and safer sexual behaviour by youths.

## Methods

### Ethics Statement

This study uses secondary data sources, and we have obtained permission of the Uganda Ministry of Local Government to carry out these analyses.

### Data

The data were collected using multiple cross-sectional district level community surveys during 2003–2010. In 2003, the Uganda AIDS Commission (UAC), with the support of The World Bank, introduced Lot Quality Assurance Sampling (LQAS) methodology to monitor HIV-related indicators, including condom use, at the district and sub-district level. It was introduced during 2003 into 19 (of the then 56) districts comprising the country, and expanded to 11 more in 2004 [30], [31]. In 2009, USAID provided funding to support the roll out of LQAS as a national health sector monitoring system (http://www.starelqas.ug). This same funding will make the dataset available for wider use by the public during 2014 through an as yet unidentified Ugandan institution. By 2011, LQAS surveys had been completed in 53 of the current 112 districts comprising the country. Many of the districts implemented surveys at one or more time points from 2003–2011. During this period Uganda had sub-divided its districts leading to an increase from 56 (2003) to 112 (2011). Between 2003 and 2010 five multi-district surveys were completed and included in this study (Table 1). Areas surveyed during the first three time points were distributed across the four Uganda regions (Central, Eastern, Northern and Western), while recent time points focus on districts in the Eastern (2009, 2010) and South Western (2010) regions. All districts supported by donors were included in the study. They include The World Bank (2003–2006) and USAID (2009-present). Donors tended to select the districts they worked in, choosing those with the lowest health indicators. All data used in this study are representative of these districts, and are not intended to be representative of the country as a whole. At each time point, teams interviewed up to six respondent groups: youths 15–24 years, women 15–49 years, men 15–54 years, mothers with infants 0–11 months, mothers with children 12–23 months, and orphans and other vulnerable children. This study focuses on the analysis of data collected among youth aged 15–24 interviewed between 2003 and 2010 for which the measures of condom usage and sexual behaviour remained unchanged.

**Table 1:** Overview of samples of youths included in this study across time and region in Uganda during 2003 to 2010.

Year	Region	TotalNo. ofdistricts	Total No. ofdistrictssurveyed	Total No.sampled	No. responses forCU at firstintercourse	No. responses forCU at most recentintercourse	No. responses for CUat lastintercourse withNR partner	No. responsesfor frequencyof CU
2003	Central	13	6	612	428	406	85	426
	Eastern	15	5	488	323	303	41	321
	Northern	13	2	297	174	167	19	172
	Western	15	6	659	380	366	41	379
	All	56	19	2056	1305	1242	186	1298
2004	Central	13	1	283	172	180	44	172
	Eastern	15	4	190	153	155	59	153
	Northern	13	4	190	126	132	32	126
	Western	15	2	380	253	260	86	251
	All	56	11	1043	704	727	221	702
2006	Central	13	3	284	175	176	22	174
	Eastern	15	2	190	96	94	21	95
	Northern	13	2	223	148	147	20	147
	Western	15	5	490	318	317	56	314
	All	56	12	1187	737	734	119	730
2009	Eastern	24	4	406	250	236	35	253
	All	80	4	406	250	236	35	253
2010	Eastern	32	9	937	580	537	118	582
	Western	30	14	1558	650	589	84	648
	All	112	23	2495	1230	1126	202	1230
Total				7187	4226	4065	763	4213

CU: Condom use.

NR: Non-regular.

### Sampling Methodology

The LQAS methodology was used at all five time points included in this study. The method first appeared in the context of industrial quality control [32]. During the mid-1980s it was adapted to the health sciences [33], [34]. LQAS is a classification method conducted at a local level to assess administrative units according to a coverage target for an intervention. Typically, a manager of a *catchment area (CA)* is trained to use LQAS to assess each administrative unit or *supervision area (SA)* responsible for implementing the intervention. In Uganda, districts (the CAs) are divided into counties and then further divided into sub-counties (the SAs). In Uganda, LQAS was introduced to classify the SA. An in-depth review of LQAS can be found in [33]. However, this paper makes use of another feature of the survey design. Each district surveyed was stratified into 4 to 7 SAs, based upon how district teams delivered services. In each SA, a sample of 19 random locations, using villages (or 24 if 4 SAs) were selected using probability proportional to size (PPS) sampling. In each location, individuals were randomly selected using segmentation sampling. As The SA sample size was selected so that when county data are aggregated, the resulting district-level coverage proportion estimates for key indicators may be calculated with a 95% confidence interval not exceeding ±10% [35], [36]. When multiple district data are aggregated the range of the confidence interval narrows substantially. As this paper does not make use of LQAS classifications we refer the reader to other sources for further discussion of the technique [33], [34], [37].

### Data Collection

Data were collected using a pretested, structured household questionnaire. Questionnaires were conducted with eligible respondents: either male or female, aged 15–24 years old. If more than one candidate was present in the household then one was selected randomly. Data collectors were health professionals from the county and district they were responsible for managing.

### Measures

Each survey included questions on social demographic characteristics of the youth, HIV Counselling and Testing (HCT) history, sexual behaviour and prevention of mother to child transmission of HIV (PMTCT), and sexually transmitted disease (STI), and HIV knowledge. Condom usage, referring to male condom usage only, was measured with five questions:

1. *‘Have you ever used a condom during sexual intercourse?’*
2. *‘Did you use a condom the first time you had sexual intercourse?’*
3. *‘The last time you had sexual intercourse did you use a condom?’*
4. *‘In the last act of sexual intercourse with a person who is not your wife (husband) or someone you live with or a regular partner did you use a condom?’*
5. *‘When you have sexual intercourse, do you always use a condom or only sometimes or never?’*

The first four questions had response options Yes/No. The last question was used to define the variable ‘Always uses a condom (Yes/No)’. Variables selected as potential factors were sex, age, marital status, education level, age at first sexual intercourse, perceived risk of HIV, ever had an HIV test, ever had an HIV test and knows the result, survey year and Ugandan region. Table 1 shows the breakdown of sample sizes for our measures of primary interest at the different time points and Ugandan regions surveyed.

### Data Analysis

Data from the five time points were merged into one database and analysed in R-v2.13.1. The analysis was restricted to individuals who reported any history of sexual activity. A sexually active participant was defined as anyone reporting age at first sexual intercourse as between 10 and 24 years, anyone reporting ever using a condom, anyone having intercourse with a non-regular partner (NR) in the last 12 months, anyone ever been married (currently married, widowed or divorced/separated), or anyone not captured with the previous four criteria who answered Yes to the question ‘*Have you ever had sexual intercourse*?’ We first estimated the effect of condom use at first sex on condom use at the most recent sexual encounter, condom use with a NR partner and consistency of condom use. The associations were later adjusted for potentially confounding factors. Variables were selected as potential confounders if they were significantly associated (p<0.1) with any of the condom usage measures. All associations were measured using logistic regression. Significance was assessed with likelihood ratio tests and estimates reported with 95% Wald confidence intervals.

## Results

Of the 7187 youths included in the study across the five time points, 63% (n = 4518) reported a history of sexual activity. This corresponds with the 2011 DHS, which found 64% of Ugandan youths to be sexually active (UDHS, 2011). This subset was used for the analysis; their descriptive data are shown in Table 2. Sexually active youths were almost equally divided between females (50.3%) and males (49.7%). Their average age was 20.2 years within a range of 15–24 years. Most individuals (74.8%) had their first sexual intercourse between the age of 15 and 19 years, mean 16.5, median 17.0. Approximately 45% were married or lived with their partner. Almost 35% had attended secondary or post-secondary level education. A third (33.1%) of the participants perceived themselves as being at high risk of acquiring HIV. Almost 60% of participants reported having ever used a condom; however, only 34% and 30% reported using a condom at sexual debut and during their most recent sexual intercourse, respectively. More than 45% of the participants who reported having intercourse with a NR partner in the previous year used a condom at last intercourse. Nearly 19% of sexually active youths reported always using a condom during intercourse, while, alarmingly, almost 49% reported never using one.

**Table 2:** Characteristics of sexually active youths participating in LQAS surveys across Uganda between 2003 and 2010 (n = 4518).

Characteristic	Number/Denominator*		Percentage
*Socio-demographic*			
**Sex**			
Male	2235/4493		49.7%
Female	2258/4493		50.3%
**Age (years)**			
15–19	1756/4518		38.9%
20–24	2762/4518		61.1%
Mean; median	20.2; 20.0		
**Age at first intercourse (years)**			
10–14	689/4269		16.2%
15–19	3194/4269		74.8%
20–24	386/4269		9.0%
Mean; median	16.5; 17.0		
**Marital Status**			
Single (No partner/Non-regular partner/Regular partner)	2365/4513		52.4%
Married or living together	2021/4513		44.8%
Widowed/Divorced/Separated	127/4513		2.8%
**Education**			
None	278/4483		6.2%
Primary (Incomplete & Completed)	2651/4483		59.1%
Secondary (O level, A level)	1347/4483		30.0%
Post-secondary	147/4483		4.6%
*Perception and attitudes related to HIV*			
**Perceived risk of acquiring HIV**			
None	1155/4507		25.6%
Low	1374/4507		30.5%
High	1490/4507		33.1%
Don’t know	482/4507		10.7%
Known HIV positive status**	6/4507		0.1%
**Ever had an HIV test**	1329/4518		29.4%
**Ever had an HIV test and knows result**	1172/4518		25.9%
*Condom usage*			
**Ever used a condom**	2652/4431		59.9%
**Used a condom at sexual debut**	1432/4226		33.9%
**Used a condom at most recent intercourse**	1218/4065		30.0%
**Had intercourse with a NR partner in last 12 months**			
Yes	1226/4117		29.8%
No	2891/4117		69.5%
No response	45/4117		1.1%
**Used a condom at last intercourse with NR partner**	566/1226		46.2%
**Frequency of condom use**			
Always	789/4213		18.7%
Sometimes	1368/4213		32.5%
Never	2056/4213		48.8%

*Denominators may vary due to missing data.

**Response option only present for 12 districts surveyed in 2010.

### Logistic Regression Models

Table 3 shows the univariate regression of condom use behaviour outcomes on selected independent variables. The main behaviour outcomes of interest include: condom use at sexual debut, condom use at most recent sexual intercourse, condom use at last intercourse with a NR partner and frequency of condom use.

**Table 3:** Odds ratios of the effects of socio-demographic and behavioural characteristics on condom use and sexual practice.

	Condom use at firstintercourse (n = 4226)	Condom use at mostrecent intercourse(n = 4065)	Condom use at lastintercourse with NRpartner(n = 763)	Always uses acondom (n = 4213)
**Univariate Models**				
Age (years)	0.96(0.94–0.99)***	0.95(0.93–0.98)***	1.06(0.99–1.13)*	0.94(0.91–0.97)***
Sex (ref: Male)	0.95(0.84–1.08)	0.53(0.46–0.6)***	0.86(0.61–1.21)	0.47(0.4–0.55)***
Education (ref: None)	***	***	***	***
Primary	1.51(1.24–1.83)	1.62(1.31–2.02)	1.21(0.76–1.9)	2.32(1.72–3.12)
Secondary	3.48(2.87–4.23)	4.03(3.25–5)	3.46(2.1–5.68)	6.23(4.67–8.32)
Post-Secondary	4.47(3.07–6.52)	6.12(4.16–9.01)	3.85(1.39–10.7)	8.34(5.37–12.93)
Married or living together (ref: Other)	0.47(0.41–0.54)***	0.15 0.12–0.17)***	0.66(0.45–0.96)**	0.09(0.07–0.12)***
Age at first intercourse (years)	1.09(1.06–1.12)***	1.02(0.99–1.05)	1.14(1.06–1.22)***	1.03(1–1.07)*
Region (ref: Central)	**	**	**	**
Eastern	0.68(0.57–0.81)	0.6(0.5–0.73)	0.32(0.19–0.55)	0.58(0.47–0.71)
Northern	0.46(0.37–0.58)	0.51(0.4–0.64)	0.21(0.12–0.38)	0.49(0.37–0.65)
Western	0.41(0.34–0.5)	0.51(0.42–0.62)	0.48(0.27–0.84)	0.48(0.39–0.6)
Survey Year (ref: 2003)	***	***	***	***
2004	1.06(0.87–1.28)	1.05(0.86–1.27)	0.09(0.05–0.17)	0.98(0.78–1.22)
2006	1.28(1.06–1.54)	1.17(0.96–1.42)	0.96(0.41–2.2)	1.04(0.83–1.29)
2009	1.37(1.04–1.8)	1.01(0.75–1.37)	0.13(0.06–0.31)	0.92(0.65–1.28)
2010	0.77(0.65–0.91)	0.71(0.59–0.85)	0.26(0.14–0.48)	0.55(0.45–0.68)
Perceived risk of HIV (ref: none)	**	***		***
Low risk	1.1(0.93–1.31)	1.2(1–1.44)	0.94(0.57–1.54)	1.03(0.84–1.26)
High risk	1.02(0.86–1.21)	0.92(0.77–1.1)	0.67(0.41–1.08)	0.78(0.63–0.96)
Don’t know	0.74(0.58–0.95)	0.69(0.53–0.9)	0.65(0.34–1.25)	0.57(0.41–0.77)
Ever tested for HIV (ref: no)	1.21(1.06–1.39)***	1.07(0.93–1.25)	1.32(0.9–1.94)	0.99 (0.84–1.18)
Ever tested for HIV and know results (ref: no)	1.19(1.03–1.37)**	1.05(0.9–1.22)	1.38(0.92–2.07)	1.02(0.86–1.22)
Tested for HIV in last 12 months(ref: no)‡	1.23(1.01–1.5)**	1.16(0.94–1.44)	1.02(0.57–1.81)	1.21(0.95–1.55)
Tested for HIV in last 12 monthsand know results(ref: no)‡	1.12(0.89–1.41)	1.17(0.91–1.49)	0.94(0.49–1.82)	1.24(0.94–1.64)
Condom use at first intercourse (crude)	–	10.37(8.87–12.11)***	4.19(2.93–5.99)***	11.93(9.89–14.39)***
Male only	–	10.91(8.83–13.49)***	5.32(3.36–8.42)***	11.59(9.18–14.63)***
Female only	–	11.24(8.8–14.36)***	2.91(1.6–5.28)***	15.61(11.04–22.07)***
**Multivariate Model**				
Condom use at first intercourse(adjusted) †	–	9.63(8.03–11.56)***	3.48(2.27–5.33)***	11.12(8.95–13.81)***
Male only ††	–	11.08(8.65–14.18)***	4.56(2.63–7.91)***	11.10(8.53–14.46)***
Female only ††	–	8.41(6.35–11.14)***	2.33(1.11–4.9)**	11.06(7.51–16.28)

NR: Non-regular.

Ref: The reference group for the Odds Ratio calculation.

*Variable is statistically significant at p<0.1.

**Variable is statistically significant at p<0.05.

***Variable is statistically significant at p<0.01.

‡ Data collected in 2006, 2009, 2010 only.

† Controlled for sex, age, education, marital status, age at first intercourse, region, survey year, perceived risk for HIV and ever tested for HIV.

†† Controlled for age, education, marital status, age at first intercourse, region, survey year, perceived risk for HIV and ever tested for HIV.

For all four outcomes, condom usage increased from 2003 to 2006. We observed geographic disparities. For all four outcomes, condom use was significantly higher in the Central region. The odds of condom use at sexual debut, at last intercourse and consistent condom use were respectively, 48%, 18% and 18% higher in the Eastern than in the Northern region. The odds of condom use at last intercourse with a NR partner were 50% lower in the Northern than in the Western region. The odds of condom use at first intercourse were 66% higher in the Eastern than in the Western region. These results should be interpreted with caution, since the surveyed geographical area varies between time points. In particular the 4 districts surveyed in 2009 were located in the Eastern region, while the 2010 survey included 9 additional districts in the Western region.

The results also show that the odds of using a male condom at last intercourse or consistently during sexual intercourse are, respectively, 47% and 53% lower for women than for men. However there is no significant difference in the odds of condom use at first sex between males or females. The odds of condom use at sexual debut, most recent intercourse or consistent condom use during sexual intercourse decrease by about 5% for every year increase in the respondent’s age.

Additionally, for every one-year older a youth is when they first have intercourse, the odds using a condom at their sexual debut increases by almost 10%. Compared to their single counterparts, the odds of using a condom at last intercourse or at sexual debut are respectively 85% and 53% higher among youth who are married or living with their partner. Furthermore, youth who are married or living with their partner have a 91% increase in the odds of consistently using a condom compared to those who do not live with a partner or a spouse. Among those who had sexual intercourse with a NR partner in the last 12-months, the odds of using a condom at last intercourse are 34% lower among youth who are married or living with their partner compared to their single counterparts.

Higher education level is strongly associated with increased condom usage. Among youths with post-secondary level schooling compared with no schooling, the odds of using a condom at sexual debut are 4 times higher, the odds of condom use at last intercourse are 6 times higher, the odds of condom use at last intercourse with a NR partner are almost 4 times higher while the odds of using a condom every time they have sex are over 8 times higher. Additionally, the odds of having used a condom at first intercourse, last intercourse and consistently are, respectively, 26%, 31% and 43% lower among youths who do not know their risk of acquiring HIV, compared to those who perceive no risk of acquiring HIV.

In line with previous studies, this study shows that condom use at sexual debut is highly associated with condom use at most recent sexual encounter. When controlling for sex, age, education, marital status, age at first intercourse, region, survey year, perceived risk for HIV and ever tested for HIV, the odds of condom use at last intercourse are almost ten times higher if a condom was used at sexual debut. Using a condom at first sex also increases by more than three the odds of condom use with a NR partner and by more than eleven the odds of consistent condom use (Table 3 and Tables S1, S2, S3 in File S1). The associations are also high when measured for males and females separately.

## Discussion

We studied the association between using a condom during first sexual intercourse and subsequent condom use in Ugandan youth in a multiple logistic regression model. Our findings show that condom usage at sexual debut is strongly correlated with condom use at most recent intercourse and with consistent condom use with both regular and NR partners. These relationships persist when adjusting for geographic region, educational level, sex, marital status, perceived risk of HIV and having ever taken an HIV test. These findings substantiate the need for youth HIV prevention and sexual health programmes introduced prior to the initiation of sexual intercourse. Encouraging youth to use condoms at first sexual encounter may help to promote and reinforce healthy sexual behaviour that lasts into later life.

In our study, we found that youth in Uganda who used a condom the first time they have sexual intercourse were nearly ten times as likely to use a condom at their most recent sexual encounter and nearly five times as likely to use a condom at last intercourse with a NR partner. This finding is consistent with studies in the United States [26], [38], Latin America [39], sub-Saharan Africa [10], [28], and Europe [40] showing that early use is associated with subsequent use. To the best of our knowledge, the current study is the first to isolate the important positive association of condom use at sexual debut even with high-risk sexual behaviours (such as sex with non-regular partners), and to demonstrate that this relationship is consistent across multiple time points and over multiple geographical locations.

One theory potentially explaining this association is that when youth understand the benefit of condom use early enough to embrace protective behaviour at the first sexual encounter, this keystone event may have a lasting impact on subsequent encounters, regardless of their type of partner. However, it may simply be the case that a certain portion of individuals have a tendency towards more healthy or protective behaviours and others may be risk takers, explaining why the same individuals maintain the health protective behaviours from the outset. Nonetheless, the multiple independent variables included in the Multivariate Model (Table 2) suggest that reality is complex and that controlling for variables such as education, age, and marital status is relevant to understanding this behaviour. Future studies will have to consider the motivations behind condom use at first and subsequent sexual acts; and need to take into account that not all sexual encounters are consensual [41].

Older age at sexual debut is associated with condom usage, regardless of partner type [28], suggesting that delaying the age of sexual debut could be encouraged as a means to reduce the risk of youths to HIV infection and unwanted pregnancy. This situation is compounded by the known increased biological vulnerability of younger women to the HIV virus. Comprehensive risk reduction programmes can include components on delaying sexual debut alongside the promotion of other behaviours that reduce risks of pregnancy and STIs, including negotiation and communication skills and correct condom use for when sexual activity does occur. This approach differs from programmes that promote abstinence alone, which often focus exclusively on abstinence from sex until marriage [42], [43]. This distinction is important considering that our study shows two thirds of Uganda youth 15–24 are already sexually active and of those, three quarters (74.8%) had their first sexual encounter between the age of 15 and 19 years old. Our study adds to a growing body of evidence suggesting that efforts should be made to promote condom use and other health protective mechanisms prior to the earliest age that youths tend to initiate sexual activity [27], [44]. Our results also suggest that such programmes can have an enduring and beneficial effect regardless of where or with whom they are implemented.

As we found that married youths were less likely to use condoms when having sex with a NR partner, prevention programmes may need to reinforce the importance of condom use for all youths. Regardless of partner type (marital or casual), there is a strong association between educational level and condom usage. Education may affect sexual behaviour in a number of ways, including improving social opportunities and access to health promoting resources that positively influence other personal characteristics known to be associated with condom use, such as self-efficacy and ability to negotiate.

Because the efficacy of condoms in protecting from HIV and STIs drops sharply when condom use is inconsistent, consistent condom use is the desirable health behaviour. One of our strongest findings is that youth in Uganda who use a condom the first time they have sexual intercourse were nearly twelve times as likely to report always using a condom. Earlier research found that condom use is a habitual action based on a learned routine, rather than being a mediated, calculated or rational decision, or one based on social or peer influences [27]. This result suggests that consistent condom use by youths is a habit, rather than a discreet decision made at each sexual encounter based on peer pressure or their knowledge of the risks of unprotected sex. A number of related studies has found that negative experiences with condom use in early sexual experiences is negatively associated with later condom use by both sexes [6], [21], [44], [45]. The combined evidence from these studies suggests that the early sexual experiences are formative. As our results show no difference between males and females in the odds of condom use at first sex, social programs should be broadly conceived and focus equally on both genders. If condom use occurs “successfully” in the encounter, it can establish behaviour that is carried forward, regardless of other social and demographic factors. These potentials are especially important in light of recent multi-country models of HIV transmission that indicate that extra-couple relations are a principal route for HIV transmission in low resource settings [46].

Even with ARV treatment options and the increased protection male circumcision may offer, early and consistent condom use is still necessary, and accrues health benefits in addition to reduced HIV transmission, such as the prevention of other STIs, and unwanted early pregnancies. In a meta-analytic review, early exposure to condom promotion messages has not been found to increase the overall frequency of sexual activity or number of partners [29]. Furthermore, when provided to higher risk groups, it has been shown to have greater potential to reduce partners and increase treatment seeking behaviour, especially when delivered as part of a comprehensive risk reduction programme [29], [43]. In addition to our evidence of the strong habit-shaping role that early condom use may play, studies in multiple settings indicate that promoting self-efficacy and negotiation skills increases condom use, particularly in females [13], [24], [47]–[50]. As a preventive action that depends on male cooperation, negotiation and self-efficacy, promotion strategies should focus on the different needs of both sexes. Confidential gender and youth friendly outlets can link voluntary testing and counselling services with the provision of free condoms and promote condom use through existing community institutions frequented by youth, whether they be faith based organisations, schools and youth centres or through the use of innovative social media. Policies, of course, should also articulate a supportive legal frameworks, as well as protective procedures, for young women who experience sexual violence. Targeted programmes have been more effective than less client-focused condom promotion efforts [29], [51], [52] but they may need more clear targeting for vulnerable youths, such as those who are out of school or internally displaced.

### Limitations

This study measures associations, not causality. The relationship between condom use at first sexual encounter is statistically correlated with more consistent condom use and with use at most recent sexual encounter with both regular and NR partners. However, we cannot show that condom use at early sex will lead to condom use at later sexual encounters. Measuring condom use at a population or individual level is limited in itself, especially without directly observing the act [53]; self-reporting bias in questionnaire data could be improved by further refinements to questionnaires and improved interviewing techniques, but not removed entirely. These data could also be subject to recall bias, and thus the association we report between condom use at sexual debut and recent condom usage might be exaggerated. Qualitative research on perceptions, motivations and social and structural barriers to condom use in the youth population in Uganda could enhance our data and contribute to the design of effective, targeted risk reduction programmes. We also do not have information on forced sexual debut. Given the high prevalence of partner violence in some areas of Uganda [54] we recognize this limitation. Also, we note the lack of data concerning the social economic status of respondents. As a result we could not include this variable in the analysis. Finally, a complex sampling design was not incorporated into the statistical analysis, as the PPS sampling weights could not all be retrieved for some of the time points and districts. This non-inclusion may have biased our estimated OR and standard errors. However the strength of the associations and large sample size suggest the influence of not incorporating the weights is mild. Imputing the missing weight values and how best to address the change in district boundaries require more methodological statistical investigation which is part of our future work.

### Conclusions

Our findings add to a growing body of evidence suggesting that young adolescents should be introduced to comprehensive HIV prevention and risk-reduction messages. Our main findings lead us to recommend promoting condom use to young people prior to the initiation of sexual activity [10] and, in particular, prior to sexual debut. If delivered successfully, these messages may help to establish a lasting and safe behaviour pattern in young people with benefits that extend beyond their early youth.

More research on the individual barriers to condom use could be helpful in targeting programs and identifying at-risk sub-groups who remain resistant or lack the necessary resources or power to adopt early and continuing condom use. Among needed research is replication of the research design reported here, but in other countries. Although the multi-time point, multi-location results suggest the generalizability of our result in Uganda, questions remain about its generalizability to other countries.

The findings of this study show that early condom use at sexual debut is strongly associated with later and consistent condom use in Uganda, and therefore support the development and implementation of evidence-based condom use strategies for youths prior to the initiation of sexual activity. While these results should influence educational and social policy in Uganda as a means of protecting youths, more aptly they may suggest increasing focus on condom use from the first sexual encounter both within and outside of Uganda.

## Supporting Information

File S1 **Tables S1–S3. Table S1. Adjusted odds ratios from multiple logistic regression (all sexually actives). Table S2. Adjusted odds ratios from multiple logistic regression (sexually active males). Table S3. Adjusted odds ratios from multiple logistic regression (sexually active females).**
(DOCX)
