# Supplementary material for: Putting the C Back into the ABCs: A Multi-Year, Multi-Region Investigation of Condom Use by Ugandan Youths 2003–2010
Source: PLoS One. 2014 Apr 4;9(4):e93083. doi: 10.1371/journal.pone.0093083 (PMC3976401; doi:10.1371/journal.pone.0093083)
Supplement: File S1 — Tables S1–S3. Table S1. Adjusted odds ratios from multiple logistic regression (all sexually actives). Table S2. Adjusted odds ratios from multiple logistic regression (sexually active males). Table S3. Adjusted odds ratios from multiple logistic regression (sexually active females). (DOCX) [file pone.0093083.s001.docx]

**Putting the C back into the ABCs: A multi-year, multi-region investigation of condom use by Ugandan youths 2003-2010**

**Supporting Information – Supplementary Result Tables**

Table S1 – Adjusted odds ratios from multiple logistic regression (all sexually actives) †

| Factors | Condom use at most recent intercourse | Condom use at last intercourse with NR partner | Always uses a condom |
| --- | --- | --- | --- |
| Condom use at first intercourse |  |  |  |
| No | Ref | Ref | Ref |
| Yes | 9.63(8.03-11.56)*** | 3.48(2.27-5.33)*** | 11.12(8.95-13.81)*** |
| Age (years) | 1.01(0.97-1.05) | 1.04(0.95-1.14) | 0.99(0.94-1.04) |
| Sex |  |  |  |
| Male | Ref | Ref | Ref |
| Female | 0.56(0.47-0.68)*** | 0.78(0.5-1.22) | 0.47(0.38-0.58)*** |
| Education |  |  |  |
| None | Ref | Ref | Ref |
| Primary | 1.04(0.67-1.6) | 0.89(0.37-2.15) | 1.95(1.04-3.65)** |
| Secondary | 1.57(1.01-2.45)** | 2.49(0.98-6.33) | 3.09(1.64-5.81)*** |
| Post-Secondary | 1.85(1.07-3.21)** | 2.3(0.6-8.84) | 3.52(1.72-7.19)*** |
| Married or living together |  |  |  |
| No | Ref | Ref | Ref |
| Yes | 0.16(0.13-0.2)*** | 0.79(0.48-1.27) | 0.11(0.09-0.15)*** |
| Age at first intercourse (years) | 1(0.96-1.05) | 1.03(0.94-1.14) | 1.02(0.97-1.07) |
| Region |  |  |  |
| Central | Ref | Ref | Ref |
| Eastern | 0.87(0.66-1.14) | 0.36(0.18-0.73)*** | 0.84(0.62-1.13) |
| Northern | 0.76(0.55-1.05) | 0.33(0.16-0.67)*** | 0.72(0.5-1.04) |
| Western | 0.83(0.63-1.09) | 0.51(0.26-1.03) | 0.86(0.63-1.16) |
| Survey Year |  |  |  |
| 2003 | Ref | Ref | Ref |
| 2004 | 1.1(0.84-1.43) | 0.13(0.07-0.26)*** | 0.95(0.71-1.28) |
| 2006 | 1(0.78-1.29) | 0.88(0.36-2.18) | 0.78(0.59-1.04) |
| 2009 | 1.12(0.72-1.72) | 0.26(0.09-0.71)*** | 0.9(0.55-1.46) |
| 2010 | 0.75(0.57-0.98)** | 0.32(0.15-0.67)*** | 0.56(0.41-0.77)*** |
| Perceived risk of HIV |  |  |  |
| None | Ref | Ref | Ref |
| Low risk | 0.89(0.7-1.12) | 0.46(0.25-0.84) | 0.74(0.57-0.96)** |
| High risk | 1.28(1.01-1.61) | 0.81(0.44-1.48) | 1.02(0.79-1.33) |
| Don’t know | 0.97(0.7-1.35) | 0.88(0.4-1.94) | 0.85(0.57-1.26) |
| Ever tested for HIV |  |  |  |
| No | Ref | Ref | Ref |
| Yes | 1.3(1.05-1.61)** | 1.08(0.66-1.77) | 0.81(0.63-1.03) |
|  |  |  |  |

† Odds ratio adjusted for all variables in model; Variables included in this model were selected based on statistically significant univariate associations (p<0.05) with any of the condom usage measures.

** p<0.05, *** p<0.01.

Table S2 – Adjusted odds ratios from multiple logistic regression (sexually active males) †

| Factors | Condom use at most recent intercourse | Condom use at last intercourse with NR partner | Always uses a condom |
| --- | --- | --- | --- |
| Condom use at first intercourse |  |  |  |
| No | Ref | Ref | Ref |
| Yes | 11.08(8.65-14.18)*** | 4.56(2.63-7.91)*** | 11.1(8.53-14.46)*** |
| Age (years) | 1.01(0.96-1.07) | 0.99(0.88-1.12) | 1(0.94-1.07) |
| Education |  |  |  |
| None | Ref | Ref | Ref |
| Primary | 1.01(0.56-1.82) | 1.06(0.34-3.37) | 1.73(0.8-3.74) |
| Secondary | 1.71(0.94-3.13) | 3.16(0.96-10.45) | 2.72(1.25-5.9)** |
| Post-Secondary | 1.54(0.73-3.21) | 1.93(0.37-10.25) | 2.74(1.13-6.65)** |
| Married or living together |  |  |  |
| No | Ref | Ref | Ref |
| Yes | 0.17(0.12-0.23)*** | 0.83(0.44-1.55) | 0.15(0.1-0.21)*** |
| Age at first intercourse (years) | 1.02(0.97-1.08) | 1.03(0.92-1.16) | 1.02(0.96-1.08) |
| Region |  |  |  |
| Central | Ref | Ref | Ref |
| Eastern | 0.89(0.62-1.29) | 0.43(0.17-1.08) | 0.88(0.6-1.29) |
| Northern | 0.6(0.4-0.92)** | 0.29(0.11-0.75)** | 0.62(0.4-0.97)** |
| Western | 0.7(0.48-1) | 0.54(0.21-1.38) | 0.72(0.49-1.06) |
| Survey Year |  |  |  |
| 2003 | Ref | Ref | Ref |
| 2004 | 1.14(0.81-1.61) | 0.06(0.02-0.16)*** | 1.03(0.71-1.49) |
| 2006 | 0.87(0.62-1.22) | 0.52(0.13-2.04) | 0.68(0.47-0.98)** |
| 2009 | 0.63(0.33-1.19) | 0.08(0.02-0.32)*** | 0.8(0.41-1.53) |
| 2010 | 0.72(0.51-1.02) | 0.12(0.04-0.35)*** | 0.5(0.34-0.74)*** |
| Perceived risk of HIV |  |  |  |
| None | Ref | Ref | Ref |
| Low risk | 1.33(0.99-1.8) | 0.99(0.47-2.09) | 1.21(0.87-1.67) |
| High risk | 0.91(0.67-1.24) | 0.46(0.22-0.97)** | 0.83(0.6-1.16) |
| Don’t know | 0.89(0.56-1.41) | 0.93(0.33-2.6) | 0.88(0.53-1.46) |
| Ever tested for HIV |  |  |  |
| No | Ref | Ref | Ref |
| Yes | 1.43 (1.07-1.92)* | 0.97 (0.51-1.84) | 0.67 (0.49-0.91)* |
|  |  |  |  |

† Odds ratio adjusted for all variables in model; Variables included in this model were selected based on statistically significant univariate associations (p<0.05) with any of the condom usage measures.

** p<0.05, *** p<0.01.

Table S3 – Adjusted odds ratios from multiple logistic regression (sexually active females) †

| Factors | Condom use at most recent intercourse | Condom use at last intercourse with NR partner | Always uses a condom |
| --- | --- | --- | --- |
| Condom use at first intercourse |  |  |  |
| No | Ref | Ref | Ref |
| Yes | 8.41(6.35-11.14)*** | 2.33(1.11-4.9)** | 11.06(7.51-16.28)*** |
| Age (years) | 1.01(0.95-1.08) | 1.1(0.94-1.28) | 0.97(0.89-1.05) |
| Education |  |  |  |
| None | Ref | Ref | Ref |
| Primary | 1.1(0.58-2.1) | 0.55(0.12-2.6) | 2.48(0.81-7.57) |
| Secondary | 1.42(0.73-2.75) | 1.74(0.33-9.14) | 3.7(1.21-11.34)** |
| Post-Secondary | 2.75(1.18-6.41)** | 3.45(0.24-50.56) | 5.55(1.6-19.28)*** |
| Married or living together |  |  |  |
| No | Ref | Ref | Ref |
| Yes | 0.15(0.11-0.2)*** | 0.79(0.35-1.78) | 0.08(0.05-0.12)*** |
| Age at first intercourse (years) | 0.96(0.89-1.03) | 1.07(0.87-1.32) | 1(0.91-1.1) |
| Region |  |  |  |
| Central | Ref | Ref | Ref |
| Eastern | 0.77(0.51-1.16) | 0.21(0.07-0.63)*** | 0.72(0.44-1.17) |
| Northern | 1.04(0.62-1.74) | 0.35(0.1-1.17) | 0.87(0.46-1.65) |
| Western | 1.01(0.67-1.51) | 0.37(0.12-1.17) | 1.1(0.68-1.78) |
| Survey Year |  |  |  |
| 2003 | Ref | Ref | Ref |
| 2004 | 1.09(0.72-1.67) | 0.38(0.13-1.1) | 0.88(0.53-1.47) |
| 2006 | 1.24(0.84-1.84) | 1.72(0.47-6.38) | 1.05(0.65-1.68) |
| 2009 | 2.07(1.12-3.82)** | 1.63(0.31-8.72) | 1.31(0.61-2.79) |
| 2010 | 0.88(0.57-1.37) | 1.6(0.47-5.4) | 0.78(0.46-1.32) |
| Perceived risk of HIV |  |  |  |
| None | Ref | Ref | Ref |
| Low risk | 1.18(0.81-1.72) | 0.53(0.17-1.64) | 0.74(0.48-1.16) |
| High risk | 0.86(0.6-1.25) | 0.41(0.14-1.21) | 0.61(0.39-0.94)** |
| Don’t know | 1.03(0.63-1.69) | 0.72(0.18-2.86) | 0.78(0.41-1.45) |
| Ever tested for HIV |  |  |  |
| No | Ref | Ref | Ref |
| Yes | 1.13(0.82-1.55) | 1.72(0.73-4.04) | 1.08(0.73-1.59) |
|  |  |  |  |

† Odds ratio adjusted for all variables in model; Variables included in this model were selected based on statistically significant univariate associations (p<0.05) with any of the condom usage measures.

** p<0.05, *** p<0.01.
